# Promoting the Quality of Teacher-Toddler Interactions: A Randomized Controlled Trial of “Thrive by Three” In-Service Professional Development in 187 Norwegian Toddler Classrooms

**DOI:** 10.3389/fpsyg.2021.778777

**Published:** 2021-11-18

**Authors:** Elisabet Solheim Buøen, Ratib Lekhal, Stian Lydersen, Turid Suzanne Berg-Nielsen, May Britt Drugli

**Affiliations:** ^1^Regional Centre for Child and Adolescent Mental Health, Eastern and Southern Norway, Oslo, Norway; ^2^Department of Communication and Culture, Norwegian Business School, Oslo, Norway; ^3^Department of Education, University of Oslo, Oslo, Norway; ^4^Regional Centre for Child and Youth Mental Health and Child Welfare, Faculty of Medicine and Health Science, Department of Mental Health, Norwegian University of Science and Technology (NTNU), Trondheim, Norway; ^5^Centre of the Study of Educational Practice, Inland Norway University of Applied Sciences, Hamar, Norway

**Keywords:** childcare quality, teacher-toddler interactions, professional development, classroom assessment scoring system, cluster randomized controlled trial

## Abstract

The effectiveness of the Thrive by Three intervention, a 10-month, multicomponent, in-service professional development model to promote the quality of caregiver-toddler interactions (i.e., process quality), was tested utilizing a clustered randomized controlled design. Eighty childcare centers with 187 toddler classrooms in Norway were randomly assigned to either the Thrive by Three intervention group (*n*=87) or a usual-activity wait list control group (*n*=100). Interactional quality was assessed with the Toddler version of the Classroom Assessment Scoring System (CLASS-Toddler) at three timepoints: pre-, mid-, and post-intervention. There were significant group differences in change in quality during the intervention period in both CLASS domains, Emotional and Behavioral Support (EBS), and Engaged Support for Learning (ESL), with greater overall differences in the ESL domain. Quality increased in the intervention groups, but quality decreased in the control group from baseline to post-intervention. There were significant group differences in quality at baseline. The Thrive by Three intervention had a positive effect on teacher-toddler interactions in both the EBS and ESL domains. Results need to be replicated preferably in more diverse samples.

**Clinical Trial Registration:** ClinicalTrials.gov #NCT03879733.

## Introduction

A third of all children in OECD countries under the age of 3years attend professional childcare (OECD, 2019). In Norway, as much as 85.4% of 1- and 2-year-olds are in formal childcare, with most of them attending for long hours (Statistics Norway, 2021c). Childcare quality is of great importance for all these young children, both in the short and long run (Vandell et al., 2010; Sylva et al., 2011). Despite the importance of high-quality care, the quality of caregiver-toddler interactions in childcare centers around the world is typically found to be in the medium range (Vermeer et al., 2016). Recent evidence from Norway, the context for the present study – where universal access to highly subsidized childcare is a law-given right from the age of 1year, and where important quality parameters such as caregiver-child ratio, group size, and caregiver educational level are regulated by the state – confirms Vermeer et al. (2016) findings of medium range quality. Moreover, caregiver-toddler interactions vary in quality both between and within centers (Bjørnestad and Os, 2018; Bjørnestad et al., 2019). Several in-service continuous professional development programs have been developed to improve the quality of caregiver-child interactions (process quality; Werner et al., 2015; Egert et al., 2018). Meta-analyses and systematic reviews have found a medium effect size for enhancement of interaction quality after implementing professional development programs (e.g., Werner et al., 2015; Markussen-Brown et al., 2017; Egert et al., 2018, 2020). However, studies with sufficiently rigorous designs to study the effects of such programs on process quality are lacking for the youngest children (ages 1 to 3years) in childcare (see Eurofond, 2015; Egert et al., 2018). In an attempt to fill this void, we developed, implemented, and tested a multicomponent, professional development model, Thrive by Three, aimed at improving the quality of caregiver-toddler interactions (i.e., process quality) in 187 toddler classrooms in Norway. The model is based on the Classroom Assessment Scoring System (CLASS; La Paro et al., 2012) and previous research on professional development (Sheridan, 2001; Sheridan et al., 2009; Eurofond, 2015). It has a specific focus on competence building with all staff in the toddler classroom to foster more positive caregiver-toddler interactions.

Quality of childcare is usually defined in terms of structural quality (e.g., group size, caregiver-child ratio, and caregiver education level) and process quality (e.g., caregiver-child interactions, peer interactions, and parental involvement; Scobie and Scott, 2017). Caregiver-child interactions are found to be the most important ingredient of childcare quality for young children (Pianta et al., 2016) and are thus the focus of the Thrive by Three model and present study. High-quality caregiver-child interactions are characterized by stable interactions with sensitive and responsive caregivers, a focus on play-based activities, routines that allow children to take the lead in their own learning, support for communication and language, and opportunities to move and be physically active (Scobie and Scott, 2017). Due to the fact that structural quality parameters are highly regulated by the state in Norway, variations in quality between childcare centers are mainly due to variations in process quality.

As mentioned above, process quality can be improved through professional development. Professional development in childcare centers refers to activities that develop caregivers’ skills, knowledge, and dispositions as well as their application of this knowledge. Professional development programs typically include a didactic component (i.e., workshop, lecture, seminar, or training materials) and some form of supervision, broadly defined as mentoring, coaching, feedback, or on-site support (Brunsek et al., 2020). Based on evidence from 35 European studies, Eurofond (2015) concluded that professional development is most effective when it is integrated into the childcare center’s practice with a focus on reflection that leads to changes in practice and curricula (EUROFOND). Further, a meta-analysis of 42 studies by Egert et al. (2018) found that coaching of caregivers promoted the effectiveness of professional development programs; training was effective for caregivers with and without a Bachelor’s degree; and positive effects were found in childcare centers with different age groups (infants, toddlers, and preschool). In a later meta-analysis, Egert et al. (2020) concluded that the most effective professional development programs include multiple instructional formats combined and multiple components, such as self-reflection, feedback, modeling, and on-site coaching. Moderator analyses by Egert et al. (2018) revealed a curve linear relationship between the duration of the program (i.e., number of hours) and the change in caregiver-child interactions. Programs with a duration of 45–60h appeared to be more effective in changing caregiver-child interactions than both shorter and longer programs.

Despite this growing literature on how to improve process quality through professional development, important knowledge is lacking, especially on programs developed to improve caregiver-toddler interactions. A few very recent randomized controlled trial (RCT) studies have looked at the effects of teacher interventions on outcomes, such as language and math skills as well as problem behavior in toddlers (Bleses et al., 2020; Yazejian et al., 2020). However, most studies focusing on professional development programs have an observational design and typically include preschool aged children. In the Egert et al. (2018) meta-analysis, only one of the 42 studies from the North America was an RCT study with mixed age groups (including toddlers), and there were none with infant and toddlers only. Similarly, of the 35 quantitative studies included in the Eurofond (2015) systematic review, only two studies had an RCT design, and research on the youngest children in care was underrepresented in general. This highlights the need for well-designed professional development programs for professionals working in toddler care and for well-designed experimental studies to investigate the effectiveness of such programs on the quality of caregiver-toddler interactions.

Taking advantage of a large sample of 187 toddler classrooms with 1,561 toddlers and their caregivers, the primary aim of this paper was to examine to what extent Thrive by Three improves the quality of caregiver-toddler interactions in an intervention group compared to a wait list control group, after a 10-month intervention period.

## Materials and Methods

### Trial Design and Participants

The present study is part of the larger Thrive by Three cluster randomized clinical trial study (see Lekhal et al., 2020). Seven municipalities/city districts were invited and consented to participate – four in Eastern Norway and three in Central Norway. A total of 78 childcare centers and 187 toddler classrooms were included. Childcare centers within each municipality were recruited after the municipalities had consented to participate. Childcare centers volunteered to participate. All childcare centers in the participating municipalities were allowed to participate as long as they had at least one toddler group/classroom or a group with children aged 10–36months. However, a maximum of 16 childcare centers could participate from each municipality/city district. If more centers wanted to participate, the municipality chose those centers they decided fit the study best. The managers of the centers received an e-mail (or letter if needed) including an electronic link with the written informed consent. The leaders consented to their own participation as well as the enrollment of their center. In addition, the leaders forwarded the e-mail from the Thrive by Three study with the written informed consent to all professional caregivers at the center and parents of the children. The parents provided written consent for themselves and their children. If parents had shared custody, both parents had to consent before the child could be enrolled in the study. All participants had the right to withdraw from the study at any time and without explanation.

**Figure 1:**
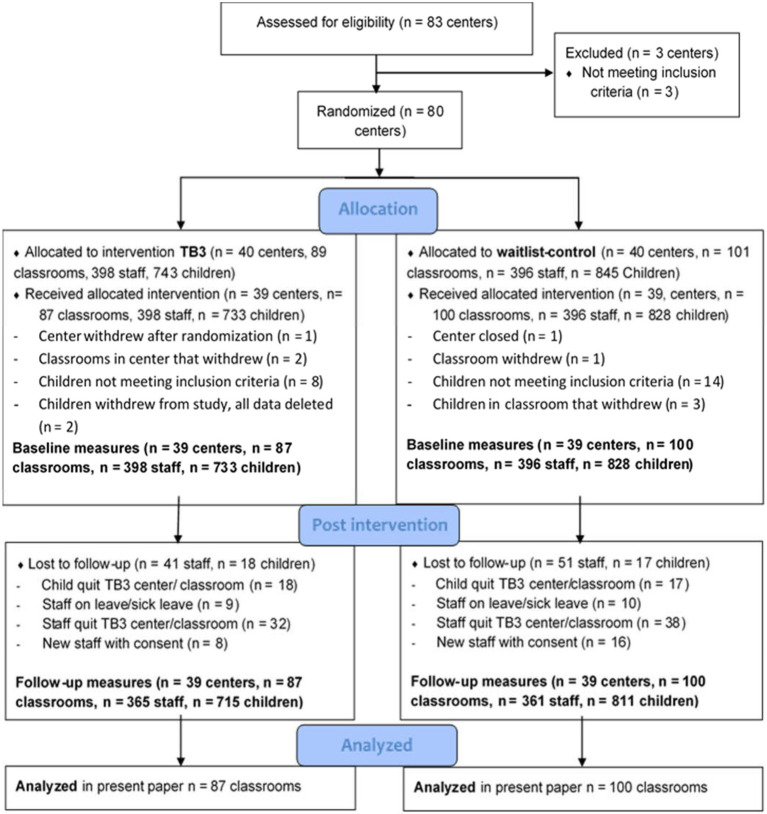
CONSORT flow diagram of recruitment, randomization, and participation of childcare centers, classrooms, staff (including leaders), and children in the Thrive by Three (TB3) study.

Figure 1 shows a flowchart of the recruitment procedure and participant rates. After recruitment, childcare centers were randomized to the intervention group or control group. Randomization was stratified within each municipality and size (small versus large) of childcare center. Within each stratum, half of the childcare centers were randomized to each of the two groups. The randomization was carried out using a random number generator by a statistician research team member who did not know the identity of the childcare centers. Childcare centers in the control group were offered participation in the intervention the year after. The present study is based on data from observations of the quality of teacher-child interactions at the classroom level. Data were collected in three waves during 1 year, before the intervention (T1 September 2018), during the intervention (T2 January 2019), and after the intervention (T3 June/July 2019). The mean age of the 1,561 children in the sample at T1 was 21.4months (SD=6.23). Girls made up almost half of the sample (48.8%). Information on ethnic background was obtained from 87.4% of the sample. Of those, most children were Norwegian (91.4%), 4.2% were from another European country or from North America, and 4.4% were from a non-Western country. Only 20 children, less than 1%, were reported to have a disability. Parents were mostly married or cohabitating (83.5%), only 3.5% were single parents, and 13% did not provide information. In Norway, relatively few households live in poverty due to a well-established social system. The parents of the children in the sample had both higher income and higher educational levels than the general population. In the sample, 87% provided information on their annual income; of these, only 4.8% had low income, compared to the national prevalence of 11.7% (Statistics Norway, 2021a). Low income was defined according to Statistics Norway as roughly under 400,000 Norwegian kroner in 2019 for Norwegian households of couples with two children. Of those who reported their educational level (87%), 75.4% of parents had a college or university degree at the Bachelor’s level (31.3%) or higher (44.1%). In the general population, 35.5% had a college or university degree. The professional caregivers were mostly women (91.3%), had worked in the classroom for an average of 3.2years (SD=3.82), and had a permanent position at the center (89.4%). Most professional caregivers were Norwegian (82.3%), 7.6% came from another European country or North America, 9.7% came from a non-western country, and 2.0% did not provide information on ethnicity. Regarding education, 34.6% had ECEC teacher education Bachelor’s degree, which is very close to the national average of 34% (Statistics Norway, 2021b), 7.6% had a Bachelor’s degree (not teacher), 2.8% had a Master’s degree, and 2.0% did not answer.

### Intervention: The Thrive by Three Model

Thrive by Three is a 10-month CLASS-based professional development program specifically aimed at increasing the process quality in toddler classrooms in Norwegian childcare centers. The Thrive by Three intervention is grounded in transactional models for development (Sameroff, 2000; Shonkoff and Fisher, 2013), research showing that interactions between young children and caregivers are a primary mechanism of child mental health, development, and learning (Sroufe, 2000; Pianta et al., 2003; La Paro et al., 2004; Burchinal et al., 2015), and research indicating that professional development programs can be effective for improving caregiver-child interactions (Sheridan, 2001; Eurofond, 2015; Moreno et al., 2015). An early version of the model was piloted at 49 childcare centers in the municipality of Sandnes, Norway (Solheim et al., 2016). An important asset of Thrive by Three is that it was developed to align with the childcare centers’ practice. We sought to limit the increase in additional resources needed, and as far as possible, the full-day seminars and supervision meetings for staff and head teachers were scheduled for already planned “non-contact time” (time away from the children). One of the main goals of Thrive by Three was for staff to utilize already scheduled non-contact time for the purpose of professional development in a much more systematic way than typically found at most centers. Thus, Thrive by Three was developed to be a professional development intervention integrated in the center’s practice that would not demand too much of the staff in terms of non-contact time or of the center in terms of finances – in order to increase the intervention’s applicability and sustainability.

Thrive by Three consists of four main components:

1. *Quality assessment and feedback*. Process quality in all participating classrooms was observed by certified observers, using a standardized observation method, the Toddler version of the Classroom Assessment Scoring System (CLASS-toddler; La Paro et al., 2012). On the day of the observation, all staff in the intervention group received feedback from the certified observers on their classroom’s scores based on the observation of eight quality dimensions of the CLASS-Toddler, showing strengths and weaknesses of adult-child interactions. CLASS observations with feedback from the certified observers were repeated three times throughout the year, in September (T1), January (T2), and June (T3). Center leaders were invited to participate in the feedback sessions.
2. *Supervision and reflection*. All staff in each intervention classroom received systematic supervision sessions with their head teacher on a monthly basis based on their score on the CLASS-Toddler and an action plan for improvement. All supervision sessions followed a specific structure outlined in a supervision manual given to all supervising head teachers. In classrooms with two head teachers, they chose themselves, which one should take on the role as supervisor. At the supervision sessions staff reflected together with their head teachers upon their own practice and interaction with the children in the group, using the CLASS dimensions as the starting point and pre-prepared reflection notes, written by the staff and handed in to the supervising head teacher prior to each supervision session. Staff in each toddler classroom met 10 times with the supervising head teacher during the 10-month intervention period. At each session, the focus was on 1–2 CLASS dimensions. The supervising head teacher, in collaboration with the staff, chose which CLASS dimensions to start with, based on the feedback from the certified CLASS observers on the CLASS profile at T1 in September. Thus, which CLASS profiles would be in focus during and between the sessions varied between classrooms depending on the supervising teacher’s estimation of the staff’s challenges. However, it was a clear premise in the Thrive by Three model that all 8 CLASS dimensions would have had a special focus at some timepoint during the intervention period, in all the participating classrooms. Between supervision sessions, all staff, including the supervising head teacher, were asked to focus on 1–2 CLASS dimensions at a time in their daily work with children and reflect upon and take notes on interactions they had with the children that could be encompassed by the focused CLASS dimensions. All supervising head teachers received 1day of extra training from the research team and a written manual on how to conduct each of the 10 supervision sessions. The supervising head teachers, in turn, received supervision from mentors (typically employed in educational psychological counseling service or other resource persons in the municipality) in groups of 5–8 supervising head teachers from centers in their municipality. The supervising head teachers met in groups 10 times together with their mentor during the intervention period. They focused on 1 or 2 CLASS dimensions at a time, agreeing among them which CLASS dimension they should focus on based on the needs of the supervising head teachers and on their experiences with supervising their staff. The mentors also followed a manual to ensure that the structure of the sessions would be as similar as possible. The mentors, in turn, met 10 times with the research team to receive supervision and coaching.
3. *Child development and mental health seminars*. All childcare employees in the seven municipalities attended three full-day seminars that focused on research-based knowledge on infant and toddler development, mental health and its risk- and protective factors, early signs of mental and developmental problems, and crucial aspects of childcare quality. Head teachers from each classroom participated in one extra meeting that focused on supervision and how to address concerns about a child to his/her parents when necessary. Center leaders were also invited to participate in these meetings.
4. *Manuals, booklets, posters, and website*. Manuals on the content and conducting of supervision were provided for head teachers and mentors. In addition, comprehensive written material was provided to parents and staff in the intervention group. Based on the Washington State Early Learning and Development Guidelines (State Washington, 2012) and CLASS-Toddler (La Paro et al., 2012), we developed seven booklets for parents, adapted to the Norwegian context, on children’s early development and on how to stimulate and support their child’s development. The booklets were translated into English, Somali, Polish, and Arabic to meet the needs of minority parents. All staff received a 30-page resource booklet with research-based knowledge on children’s transition to center care, the importance of classroom organization and routines, process quality, the process of developing high process quality, and children at risk – how to identify them and what to do. We also developed posters for each CLASS dimension with examples on how to work with the content of a given dimension in everyday interactions with a child. The posters were to be posted on the walls in the classrooms and changed according to the CLASS dimensions in focus at a given time. The teachers were also encouraged to post the posters where parents could see them, so that parents would have information on which CLASS dimensions the caregivers were focusing on at a given time. Access to the project website, where in-depth information on the study, the content of the staff resource booklet, information on CLASS, and the parent booklets could be found, was also available to parents and employees in the intervention group at www.tf3.no.

The wait list control group was observed with CLASS at T1, T2, and T3 simultaneously with the intervention group but did not receive feedback on their scores.

### Outcome Measures

#### Classroom Assessment Scoring System

Observations of process quality pre intervention baseline (T1; September/October 2018), mid intervention (T2; January 2019), and post-intervention (T3; June 2019) were assessed using the Classroom Assessment Scoring System (CLASS).

The CLASS tool is an observational measure used to assess process quality in childcare centers (La Paro et al., 2012). For Thrive by Three, the Toddler version of the tool was used. CLASS-Toddler focuses on childcare centers for children aged 15 to 36months and the interactions that occur in these settings between staff and groups of children. The tool is made up of two domains: (1) Emotional and Behavioral Support (EBS) and (2) Engaged Support for Learning (ESL). The EBS domain consists of five dimensions: Positive Climate, Negative Climate [reversed], Teacher Sensitivity, Regard for Child Perspectives, and Behavior Guidance. The ESL domain has three dimensions: Facilitation of Learning and Development, Quality of Feedback, and Language Modeling. Each dimension is rated on a 7-point scale, producing a final score for each setting – 1 and 2 represent a low score, 3 to 5 represent a medium score, and 6 and 7 represent a high score. On all the dimensions, a high score indicates high process quality, except for the dimension Negative Climate, where a low score is considered positive.

CLASS was coded by 33 trained observers. The observers were head teachers (*n*=17) and center leaders (*n*=5) from childcare centers in the intervention group, special education teachers from the municipalities (*n*=5), and research assistants (*n*=6). The research assistants only served as substitute observers. Of the 33 observers, 18 observed (55%) at all three timepoints, six (18%) at two timepoints, and nine (27%) at one timepoint. The observers were trained by a certified CLASS trainer on the research team. After training, the observers scored five videotapes of toddler classrooms and reached the required reliability criterion of 80%. Booster sessions were conducted before and during the periods of data collection to maintain reliability throughout the project period. For the observers, observations meant time away from their childcare centers, and the centers were compensated financially so that they could hire substitute teachers.

The mean number of observations per observer at each timepoint was eight (T1), seven (T2), and six (T3). All classrooms were observed for 1.5–2h in the morning. Variations in CLASS scores have been observed across the day (Thorpe et al., 2020), so it was important that all classrooms were observed in the morning on all three assessment points to minimize the effect of such variations on the results. The observers observed the classroom in a total of three loops/cycles – each 15 minutes in duration. After each loop, the observer would take a break for 10 minutes and score the loop on all eight dimensions. This procedure was repeated for all loops, and the total score on each dimension reflected the average across all the three loops. The scores were entered digitally on an iPad, and the total score was automatically generated once all scores had been entered. The domain scores were calculated as the mean of the dimension scores. The dimension Negative climate was reversed.

At each timepoint, observers would observe childcare centers within their own municipality, but if the observers were either a head teacher or a center leader, they did not observe classrooms in their own childcare center. The observers did not observe the same classroom more than once. It was not feasible to have the observers blind to the classrooms’ status in terms of control/intervention. This was due to the nature of the intervention, where the childcare classrooms in the intervention group, but not wait list control classrooms, would receive immediate feedback and a written report after the observation provided by the observer.

#### CLASS Inter-rater Reliability

A portion of the observations was double-coded at baseline (T1, 10%) and post-intervention (T3, 14%). To assess inter-rater reliability, we used a two-way mixed model regression, with the domain EBS or domain ESL as dependent variable and raters and participants as crossed random effects (Gwet, 2014). The inter-rater reliability intraclass correlation coefficient (ICC) was calculated as the between participants (classroom) variance, divided by the total variance (see Gwet, 2014). The ICC was 0.88 (T1) and 0.78 (T3) for the ESB domain and 0.91 (T1) and 0.85 (T3) for the ESL domain.

### Analyses

Sample characteristics were described using mean and standard deviations for continuous variables and absolute and relative frequencies for categorical variables. Sample characteristics are reported at the classroom level (see Table 1), because this was the level of measurement of the primary outcome measure. Independent samples t-tests and Pearson’s chi-squared tests were applied to compare control and intervention groups at baseline (Table 1). Due to the nature of the intervention, the CLASS observers were not blind to the classrooms’ condition (intervention vs. control). We therefore checked for systematic differences in the CLASS means between control and intervention group at baseline, using independent t-tests for each domain and subscale. The scores on Negative Climate were reversed in the statistical analyses.

**Table 1:** Descriptive statistics for the classrooms at baseline, mean, and SD.

	Intervention group	Control group	P value
	(*n* =81)	(*n* =81)	
Number of children in the classroom	11.07 (2.66)	10.89 (2.84)	0.820
Number of staff with teacher education	1.64 (1.60)	1.59 (1.66)	0.847
Staff stability (scale 1–5)	4.04 (0.93)	4.08 (0.69)	0.773
	(*n* =87)	(*n* =100)	
CLASS EBS	6.02 (0.59)	5.62 (0.79)	**<0.001**
PC	6.15 (0.78)	5.76 (1.00)	**0.003**
NC	6.97 (0.14)	6.92 (0.20)	**0.028**
TS	6.06 (0.71)	5.58 (1.04)	**<0.001**
RCP	5.66 (0.88)	5.07 (1.06)	**<0.001**
BG	5.27 (0.96)	4.75 (1.11)	**0.001**
CLASS ESL	3.63 (1.03)	3.08 (0.97)	**<0.001**
FLD	4.03 (1.08)	3.47 (1.02)	**<0.001**
QF	3.38 (1.23)	2.80 (1.04)	**0.001**
LM	3.47 (1.03)	2.98 (1.05)	**0.001**

The quality dimension NC is reversed. EBS, emotional and behavioral support; PC, positive climate; NC, negative climate; TS, teacher sensitivity; RCP, regard for child perspective; BG, behavior guidance; ESL, engaged support for learning; FLD, facilitation of learning and development; QF, quality of feedback; LM, language modeling. Significant differences are in bold.

To evaluate the effect of the Thrive by Three intervention on childcare quality at the classroom level, we used linear mixed models with the two CLASS domains and eight quality dimensions separately, 10 models in total. The classroom was included as a random effect, and time (pre-, mid-, and post-interventions), condition, and their interaction were included as fixed effects. The effect of Thrive by Three versus control post-intervention was estimated as the difference in change from baseline for the two conditions in terms of the coefficient of the corresponding term condition×time. Normality of residuals was checked by visual inspection of Q-Q plots. In one case, for the variable Negative Climate, the Q-Q plot showed some deviation from normality. Hence, we also performed linear mixed model analysis using bootstrapping with 2000 bootstrap samples and the bias-corrected and accelerated (BCa) method. Results were substantially the same (data not shown).

As Figure 1 shows, there were no dropouts of classrooms during the intervention period. Data on classroom quality were complete on all three measurement timepoints. Two-sided *p* values <0.05 were considered statistically significant, and 95% confidence intervals (CIs) are reported where relevant. Statistical analyses were conducted in IBM SPSS Statistics 23.

## Results

### Baseline Measures

As Table 1 shows, the intervention and control group did not differ in terms of number of children in the classroom, number of staff, number of staff with a Bachelor’s degree in early childhood education, or stability of staff. However, classrooms in the intervention group had significantly higher mean scores on all CLASS dimensions at baseline. The dimension Negative Climate had very little variance [intervention group: mean (*SD*)=6.97 (0.14), range 6–7; control group: mean (SD)=6.92 (0.20), range 6–7]. Note that the Negative Climate is the only scale where a low scores indicates better quality. This scale was thus reversed in the statistical analyses.

### Intervention Effects

**Figure 2:**
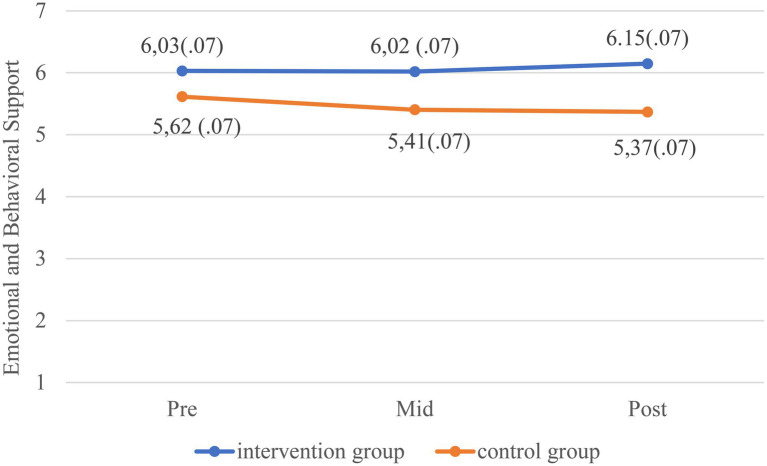
Estimated marginal means with standard errors (SE) for Emotional and Behavioral Support (ESB) at three timepoints separated by groups. Higher scores indicate better quality.

**Figure 3:**
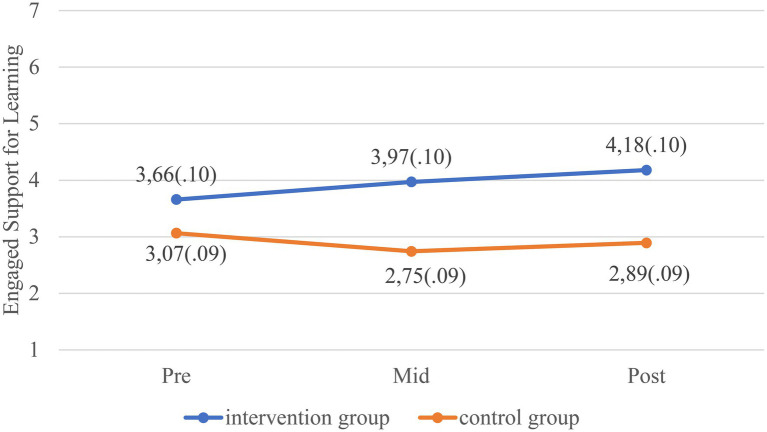
Estimated marginal means with standard error (SE) for Engaged Support for Learning (ESL) at three timepoints separated by groups. Higher scores indicate better quality.

Table 2 shows the results of the linear mixed models. The quality of the teacher-toddler interactions in the intervention group increased significantly more in both CLASS domains and all the quality dimensions from baseline to post-intervention compared to the control group, except for Negative Climate. The differences in change between the control group and intervention group from baseline to mid- and post-intervention for EBS and ESL can be seen in Figures 2, 3 (graphical displays of the changes on the dimension level can be found in Supplementary Material). Effect sizes for the EBS domain were Cohen’s d=0.3 mid intervention and 0.4 post-intervention. For the ESL domain, effect sizes were Cohen’s d=0.7 mid-intervention and 0.8 post-intervention. According to Cohen (1988), an effect size of 0.2 is considered small, an effects size of 0.5 is considered medium, and an effect size of 0.8 is considered large. For the dimensions Behavior guidance, Facilitation of learning and development, Quality of feedback, and Language modeling, the difference in quality change between the intervention and control group was evident already at mid intervention. Inspection of the means on each quality dimension at the three measurement timepoints, as well as inspection of the graphical displays, revealed that whereas the quality in the intervention groups increased during the course of the year, the quality in the control group decreased from baseline to post-intervention.

**Table 2:** Effects of Thrive by Three on CLASS domains and dimensions: results from mixed model analyses of three timepoints.

Time	CLASS domain	CLASS dimension	Intervention group (*n*=87)	Control group (*n*=100)	Difference (group*time)	P value
			Mean (*SE*)	Mean (*SE*)	Estimate (95% CI)	
Baseline	EBS		6.02 (0.07)	5.62 (0.07)		
		PC	6.15 (0.09)	5.76 (0.09)		
		NC	6.97 (0.03)	6.92 (0.02)		
		TS	6.06 (0.10)	5.58 (0.09)		
		RCP	5.66 (0.10)	5.07 (0.09)		
		BG	5.27 (0.11)	4.75 (0.10)		
	ESL		3.63 (0.10)	3.08 (0.09)		
		FLD	4.03 (0.10)	3.47 (0.10)		
		QF	3.38 (0.11)	2.80 (0.10)		
		LM	3.47 (0.11)	2.98 (0.10)		
Mid int.	EBS		6.01 (0.07)	5.40 (0.07)	0.21 (−0.03 to 0.44)	0.082
		PC	6.14 (0.09)	5.62 (0.09)	0.14 (−0.16 to 0.44)	0.368
		NC	6.97 (0.03)	6.91 (0.02)	0.01 (−0.10 to 0.08)	0.775
		TS	5.94 (0.10)	5.20 (0.09)	0.26 (−0.08 to 0.59)	0.133
		RCP	5.71 (0.10)	4.92 (0.09)	0.21 (−0.12 to 0.54)	0.219
		BG	5.30 (0.11)	4.35 (0.10)	0.43 (0.07 to 0.79)	**0.019**
	ESL		3.95 (0.10)	2.74 (0.09)	0.66 (0.33 to 0.98)	**<0.001**
		FLD	4.25 (0.10)	3.10 (0.10)	0.58 (0.23 to 0.94)	**0.001**
		QF	3.71 (0.11)	2.37 (0.10)	0.76 (0.40 to 1.1)	**<0.001**
		LM	3.89 (0.11)	2.76 (0.10)	0.63 (0.28 to 0.97)	**<0.001**
Post-int.	EBS		6.13 (0.7)	5.37 (0.07)	0.35 (0.12 to.59)	**0.004**
		PC	6.38 (0.09)	5.62 (0.09)	0.36 (0.06 to 0.66)	**0.017**
		NC	6.96 (0.03)	6.83 (0.02)	0.08 (−0.16 to.02)	0.125
		TS	6.14 (0.10)	5.23 (0.09)	0.43 (0.10 to.77)	**0.011**
		RCP	5.71 (0.10)	4.77 (0.09)	0.35 (0.02 to.68)	**0.040**
		BG	5.46 (0.11)	4.40 (0.10)	0.54 (0.18 to 0.90)	**0.003**
	ESL		4.17 (0.10)	2.90 (0.09)	0.74 (0.42 to 1.1)	**<0.001**
		FLD	4.58 (0.10)	3.23 (0.10)	0.78 (0.42 to 1.1)	**<0.001**
		QF	3.81 (0.11)	2.49 (0.10)	0.74 (0.38 to 1.1)	**<0.001**
		LM	4.11 (0.11)	2.91 (0.10)	0.71 (0.36 to 1.1)	**<0.001**

The dimension score for NC is reversed. Significant effects are in bold. EBS, emotional and behavioral support; ESL, engaged support for learning; PC, positive climate; NC, negative climate; TS, teacher sensitivity, RCP, regard for child perspectives; BG, behavior guidance, FLD, facilitation of learning and development; QF, quality of feedback; LM, language modeling.

## Discussion

Several in-service professional development programs have been developed to improve the quality of caregiver-child interactions (process quality; Werner et al., 2015; Egert et al., 2020). However, although some very recent RCT studies have looked at the effects of such interventions on child outcomes (Bleses et al., 2020; Yazejian et al., 2020), studies with sufficiently rigorous designs investigating the effects of such programs on process quality are largely lacking for the youngest children in childcare. In this study, we therefore developed and tested the effect of the Thrive by Three intervention on the quality of caregiver-toddler interactions (i.e., process quality) in toddler classrooms in Norway. Overall, the results show that Thrive by Three is an effective model for increasing the quality of caregiver-toddler interactions.

The quality of the classrooms in the intervention group increased significantly in both CLASS domains and all quality dimensions except Negative climate, from baseline to post-intervention compared to the control group. The most notable improvement was in the CLASS domain Engaged Support for Learning (ESL). For the quality dimensions within this domain Facilitation of learning and development, Quality of feedback, and Language modeling, the difference in change of quality between the intervention and control group was evident already at mid intervention. This was true for only one of the five dimensions within the Emotional and Behavioral Support domain (EBS): Behavior guidance. The lack of effect on the Negative climate dimension is probably due to low variability and a “floor effect” with most classrooms scoring 1 or 2 and confirms what has typically been found in other studies (Pakarinen et al., 2010; Broekhuizen et al., 2018). We also found that whereas the quality in classrooms in the intervention groups increased on most quality dimensions, the quality of the teacher-child interactions in the control classrooms decreased over the course of the intervention period, strengthening the conclusion that the improvement in the quality of caregiver-child interactions was due to the Thrive by Three intervention. A few studies have studied the variance in CLASS scores through the course of a typical childcare year (Buell et al., 2017; Gandhi et al., 2020) in pre-kindergarten classrooms. Although the results are somewhat inconclusive, CLASS scores are typically found to be lower in fall than spring, contrary to what was found in the control group in the present study. We can only speculate as to why the quality in the control group declined. However, one plausible interpretation may be that this is an actual indication of what happens when professional development, quality building, and collegial support are not systematically integrated into practice, underscoring the need for a model such as Thrive by Three, even just to sustain a certain level of quality throughout the course of the year.

The increase in the quality dimensions within the CLASS domain ESL was evident already mid intervention (i.e., after 3–4months), but the improvement in the EBS domain was evident only post-intervention. One obvious reason for this difference may be that classrooms were typically scored higher on the EBS dimensions than the ESL dimensions at baseline, which is a typical distribution found in other studies using the CLASS-Toddler across different countries and contexts (La Paro et al., 2014; Slot et al., 2017; Guedes et al., 2020; Bichay-Awadalla and Bulotsky-Shearer, 2021). Thus, the potential for change was much greater within the ESL dimensions. Moreover, the focus of the ESL dimensions is on increasing the child’s learning and cognitive development. As such the content of these dimensions is much more concrete regarding how the caregiver can stimulate the child, for instance by asking open-ended questions, guiding the child’s exploration, being actively involved in the child’s play, or providing specific, individualized feedback (“Wow, you have been working really hard on that puzzle”). When educated in this, the caregivers can more easily practice these skills than those “skills” needed to provide good emotional and behavioral support, such as awareness, sensitivity, warmth, and mentalizing capacity. Such qualities may be more closely linked with personality. Moreover, underlying EBS is awareness and sensitivity to child’s cues, which is more dependent upon each situation and not so much on preplanning. As such, it can be harder for teachers to improve and adjust each moment to child’s cues and interests (Norris et al., 2015). Therefore, improvements within the EBS domain may require more intensive, individualized supervision, preferably including video, over time. That being said, through supervision the staff were encouraged to focus on observable, concrete behaviors that could be improved also within the EBS domain, and one dimension within this domain, Behavior guidance, improved mid intervention as well.

The faster and greater change within the ESL domain may also in part be explained by the Norwegian context specifically. In a historical perspective, it is fairly new that so many toddlers enter childcare centers. The curriculum at the teacher colleges and universities has thus been limited in terms of the specific needs of toddlers, especially when it comes to learning and development. The focus has traditionally been more toward caretaking and children’s free play – without a specific and systematic focus on learning and development. That focus only became an explicit task for the childcare centers in 2017, when a revision of Framework Plan for kindergartens (Norwegian Directorate for Education and Training, 2017) was undertaken. Knowledge on how toddlers learn and develop, as outlined in CLASS, through the active involvement of the teacher in the child’s play, where play and learning are integrated, not separate from each other, and where the most important task for the teacher/caregiver is to support the toddler’s curiosity, exploration, and thinking by departing from the child’s own perspective and focus, is largely new to most staff at Norwegian child care centers. And much more so than the knowledge that toddlers need sensitive and caring adults who attend to their emotional and behavioral needs. As such the content of the quality dimension within the ESL domain may represent newer knowledge for the staff, providing them with a greater potential for learning and change.

### Strengths and Limitations

This study is one of the few studies that have investigated the effectiveness of a professional development model developed specifically for toddler classrooms. The large sample size of 187 classrooms and 1,561 toddlers and their caregivers is another strength of the study. As highlighted by Egert et al. (2020), the most effective professional development programs include multiple instructional formats combined and multiple components – which are also strengths of the Thrive by Three model.

It is a strength of this study that caregivers did not choose to participate or not. We randomly assigned centers to intervention or control; thus, classrooms and teachers within these centers were assigned a group depending on the center’s status. This means that the caregivers/teachers could not opt out of attending the quality assessment and feedback (component 1), supervision and reflection (component 2), or the full-day child development and mental health seminars (component 3) of Thrive by Three.

With some notable exceptions (Early et al., 2017), most studies on professional development strategies rely on teachers who have elected to participate. That type of research informs us about the benefits we might see if teachers are already invested in enhancing their practice. The results of the present study are more broadly applicable because they reveal benefits of a model of professional development for all teachers, not just teachers who are especially motivated.

It is also a strength of the Thrive by Three model that we trained childcare teachers to become observers and supervisors. In addition, the mentors supervising the teachers were selected from facilities in the municipality that were already responsible for supervising the childcare centers. We also made sure, in cooperation with the center leaders, that supervision and workshops were scheduled during already planned non-contact time. This enhances the sustainability of the model and strengthens the applicability of the findings in a real-world setting. It is also a strength of the study that quality of teacher-child interactions was measured three times during the course of the year, meaning that we investigated differences in quality change through the cycle of the year and not just pre post, as is typically done.

We recognize a number of limitations of the study. Although training teachers to be observers is a strength of the Thrive by Three model because it ensures that the competence gained through the participation in the study will be maintained in the municipalities after the end of the study, it may be a limitation in terms of methodological rigor. We found that classrooms in the intervention group had significantly higher mean scores on all CLASS dimensions at baseline, and the means, especially in the Emotional and Behavioral Support domain, were higher than what has typically been found in other samples using the CLASS-Toddler (La Paro et al., 2014; Slot et al., 2017; Guedes et al., 2020; Bichay-Awadalla and Bulotsky-Shearer, 2021). This may be due in part to the fact that the very nature of the intervention, where the intervention classrooms receive immediate feedback after the CLASS observations, made blinding impossible. Because of inexperience with the CLASS measure, especially at baseline, the observers may have felt uneasy about giving negative feedback to staff and thus may have given somewhat higher scorings in the intervention group at baseline. The means typically found in other studies are in line with the baseline scores in the control group – indicating that not being blind to the research condition may have exacerbated the scores in the intervention group somewhat at baseline. Nevertheless, because we compared the change in quality in the intervention group through the cycle of the year with the change in quality in the control group, the significant differences in quality found at post-intervention still hold.

Additionally, although the observers were certified and reliable and inter-rater reliability was good, it is impossible for independent observers to be entirely accurate and consistent in their ratings. Teacher-child interactions vary from day to day, and it is always possible that the observations took place on a particularly good or bad day. Thus, the ratings of teacher-child interactions only provide a snapshot of quality that may not be entirely exact. Finally, it should be noted that an important limitation of the present study is the use of the CLASS as both the focus of intervention and an outcome measure, a circumstance that could reflect “teaching to the test.” The lack of a separate and perhaps more independent measure of observed teacher practice is a shortcoming of the work reported here, although it is important to note that the Egert et al. (2018) review found no evidence for such bias and concluded that it is not sufficient for quality improvement to show teachers a quality rating scale, such as the CLASS, and encourage them to practice what is defined as high-quality interactions without further guidance and coaching.

### Implications for Research and Practice

As far as we know, this study is one of very few to investigate the effectiveness of a multicomponent of in-service professional development model on the interactional quality between teachers and toddlers in childcare and thus helps fill an important knowledge gap when it comes to quality building in toddler classrooms – especially in a Norwegian context. The results support Thrive by Three as an effective multicomponent model for enhancing and maintaining the quality of teacher-toddler interactions through professional development. As Thrive by Three is a newly developed model, the findings need to be replicated in further studies, with preferably more diverse samples and other cultural settings to ensure broader applicability.

## Conclusion

Thrive by Three has a positive effect on teacher-toddler interactions in both CLASS domains; Emotional and Behavioral Support and Engaged Support for Learning. Whereas quality was enhanced and sustained in the intervention classrooms through the cycle of the year, quality decreased in the control classrooms.

## Data Availability Statement

The original contributions presented in the study are included in the article/Supplementary Material, further inquiries can be directed to the corresponding author.

## Ethics Statement

The study was approved by the Regional Committees for Medical and Health Research Ethics (REK) East-South (No:2017/430). Staff, teachers and parents provided informed written consent before participation, and parents provided consent on behalf of the children. Unless one parent had sole custody, both parents needed to consent. Participants could withdraw from the study at any time, without giving any reason.

## Author Contributions

ESB, RL, and MBD wrote the first draft of the manuscript. ESB and SL carried out the analyses. TSB-N critically reviewed and commented on all drafts of the manuscript. All authors contributed to the article and approved the submitted version.

## Funding

The Thrive by Three study was funded by the Norwegian Research Council, award number 260624. The funder did not have any role in the design and conduct of the study.

## Conflict of Interest

The authors declare that the research was conducted in the absence of any commercial or financial relationships that could be construed as a potential conflict of interest.

## Publisher’s Note

All claims expressed in this article are solely those of the authors and do not necessarily represent those of their affiliated organizations, or those of the publisher, the editors and the reviewers. Any product that may be evaluated in this article, or claim that may be made by its manufacturer, is not guaranteed or endorsed by the publisher.
