# Supplementary figures and images for: Promoting the Quality of Teacher-Toddler Interactions: A Randomized Controlled Trial of “Thrive by Three” In-Service Professional Development in 187 Norwegian Toddler Classrooms
Source: Front Psychol. 2021 Nov 18;12:778777. doi: 10.3389/fpsyg.2021.778777 (PMC8637887; doi:10.3389/fpsyg.2021.778777)

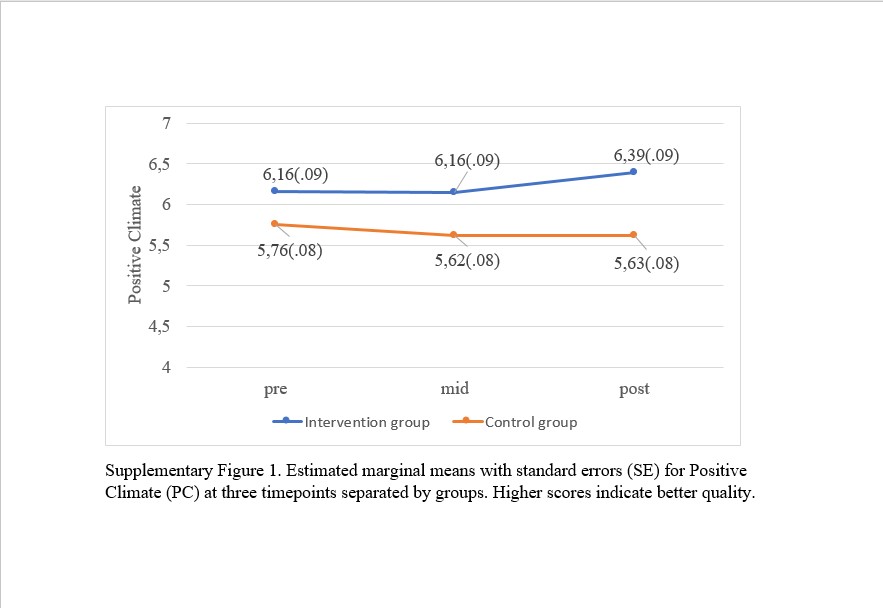

Supplement: Supplementary file 1 [file Data_Sheet_1.zip › Supplementary Figure 1_PC.jpg]

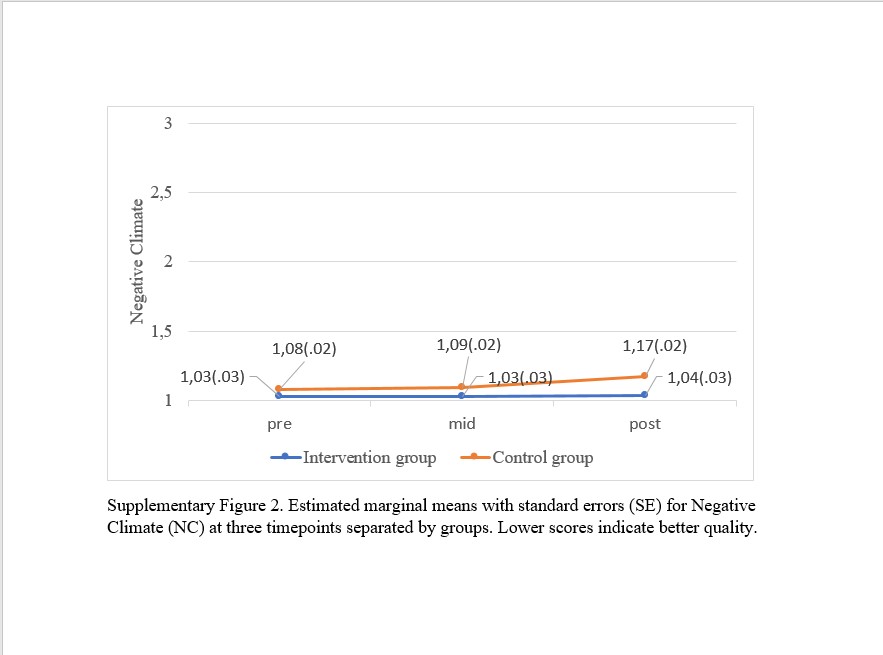

Supplement: Supplementary file 1 [file Data_Sheet_1.zip › Supplementary Figure 2_NC.jpg]

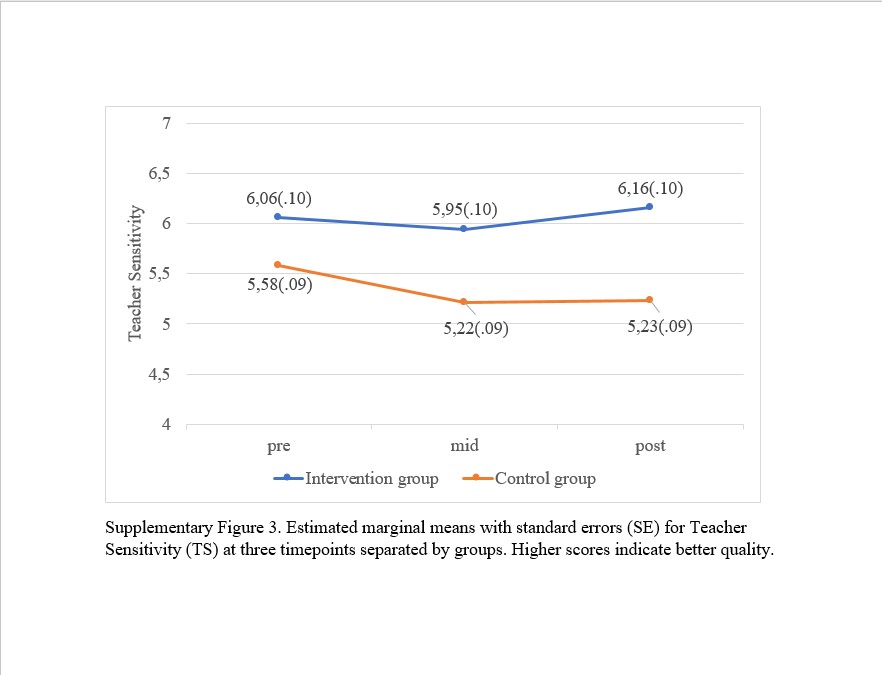

Supplement: Supplementary file 1 [file Data_Sheet_1.zip › Supplementary Figure 3_TS.jpg]

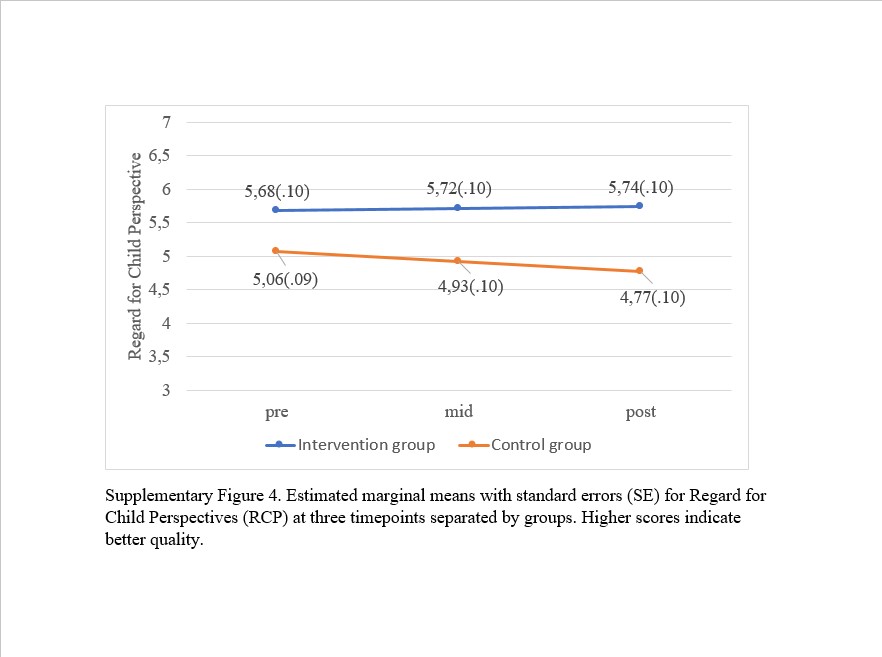

Supplement: Supplementary file 1 [file Data_Sheet_1.zip › Supplementary Figure 4_RCP.jpg]

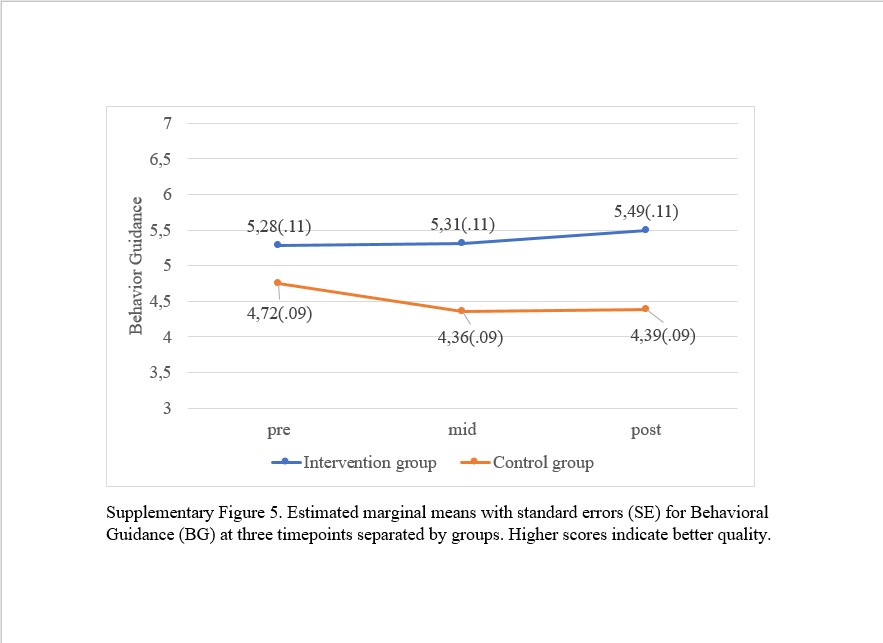

Supplement: Supplementary file 1 [file Data_Sheet_1.zip › Supplementary Figure 5_BG.jpg]

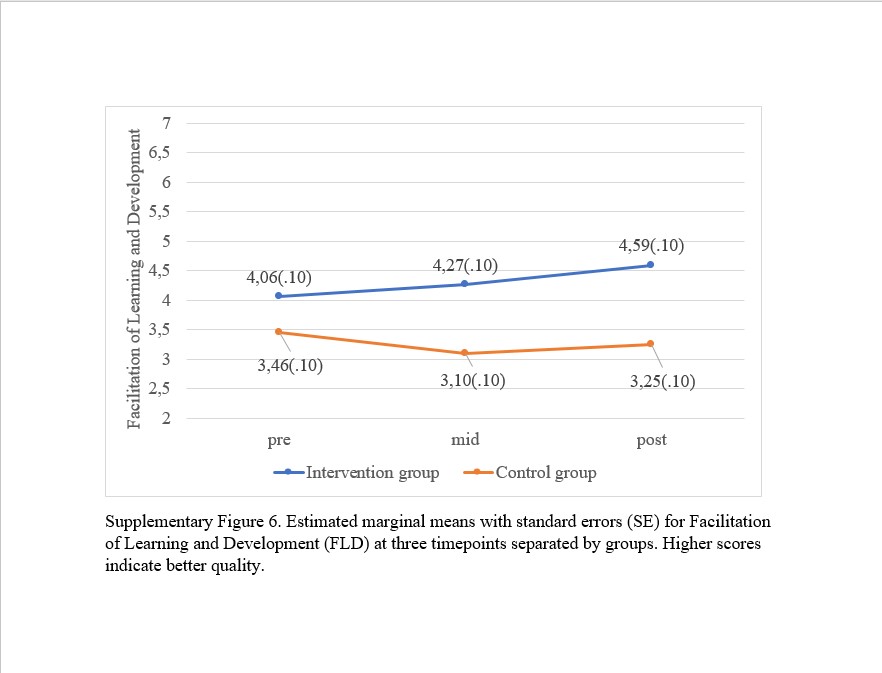

Supplement: Supplementary file 1 [file Data_Sheet_1.zip › Supplementary Figure 6_FLD.jpg]

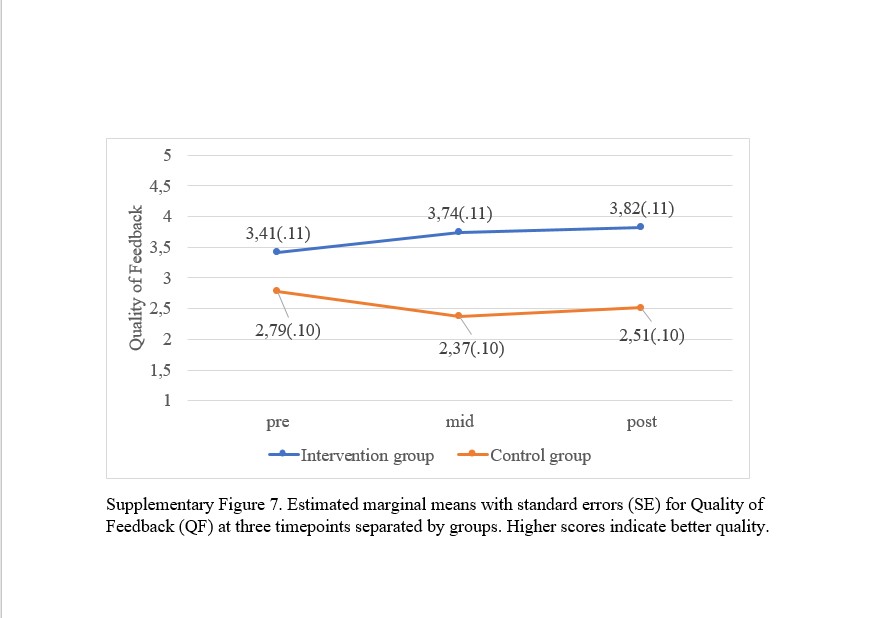

Supplement: Supplementary file 1 [file Data_Sheet_1.zip › Supplementary Figure 7_QF.jpg]

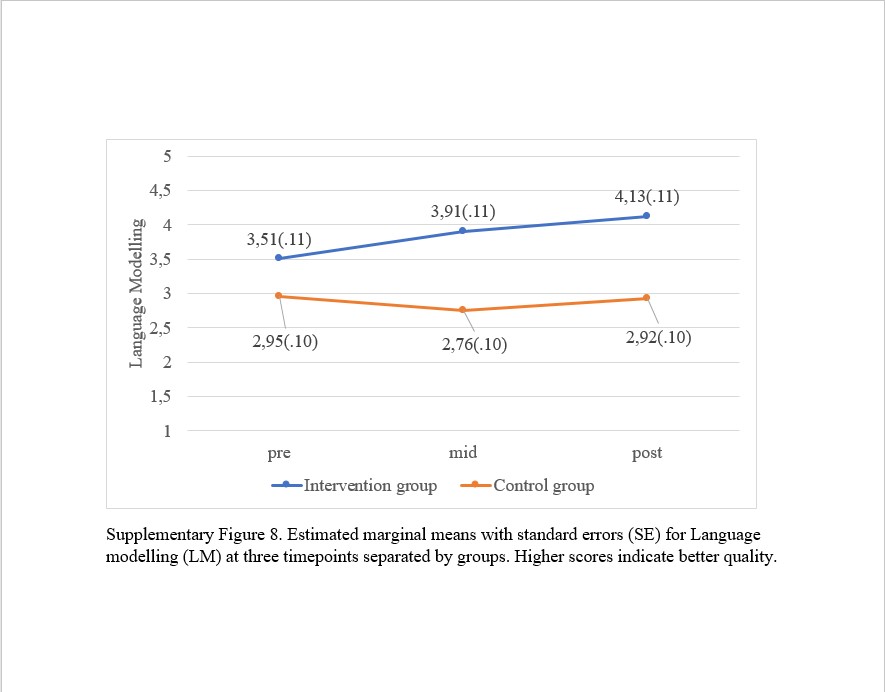

Supplement: Supplementary file 1 [file Data_Sheet_1.zip › Supplementary Figure 8_LM.jpg]
